# The Feasibility, Effectiveness and Acceptance of Virtual Visits as Compared to In-Person Visits among Clinical Electrophysiology Patients during the COVID-19 Pandemic

**DOI:** 10.3390/jcm12020620

**Published:** 2023-01-12

**Authors:** Marco Valerio Mariani, Nicola Pierucci, Giovanni Battista Forleo, Marco Schiavone, Alessia Bernardini, Alessio Gasperetti, Gianfranco Mitacchione, Mariachiara Mei, Giuseppe Giunta, Agostino Piro, Cristina Chimenti, Fabio Miraldi, Carmine Dario Vizza, Carlo Lavalle

**Affiliations:** 1 Department of Cardiovascular, Respiratory, Nephrological, Anesthesiologic and Geriatric Sciences “Sapienza” University of Rome, Viale del Policlinico 155, 00161 Rome, Italy; 2 Department of Cardiology, ASST-Fatebenefratelli Sacco, Luigi Sacco Hospital, University of Milan, 20157 Milan, Italy; 3 Cardio Thoracic-Vascular and Organ Transplantation Surgery Department, Policlinico Umberto I Hospital, 00161 Rome, Italy

**Keywords:** telemedicine, virtual visit, clinical electrophysiology, CIED, remote monitoring

## Abstract

The feasibility and effectiveness of virtual visits (VVs) for cardiac electrophysiology patients are still unknown. We aimed to assess the feasibility and effectiveness of VVs as compared to in-person visits, and to describe patient experience with virtual care in clinical electrophysiology. We prospectively enrolled patients scheduled to receive a clinical electrophysiology evaluation, dividing them in two groups: a VV group and an in-person visit group. Outcomes of interest were: (1) improvement in symptoms after the index visit, (2) disappearance of remote monitoring (RM) alerts at follow-up, (3) necessity of urgent hospitalization and (4) patient satisfaction measured by the Patient Satisfaction Questionnaire-18 (PSQ-18). This study included 162 patients in the VV group and 185 in the in-office visit group. As compared to in-person visits, VVs resulted in a similar reduction in RM alerts (51.5% vs. 43.2%, *p*-value 0.527) and in symptomatic patient rates (73.6% vs. 56.9%, *p*-value 0.073) at follow-up, without differences in urgent hospitalization rates (*p*-value 0.849). Patient satisfaction with VVs was higher than with in-person evaluation (*p*-value < 0.012). VVs proved to be as feasible and as effective as in-person visits, with high patient satisfaction. A hybrid model of care including VVs and in-person visits may become the new standard of care after the COVID-19 pandemic is over.

## 1. Introduction

In recent years, advancements in digital technology and telecommunication resulted in a tangible transformation of health care system organization and in the modality of health care delivery. The adoption of remote monitoring (RM) for the follow-up of cardiac implantable electronic device (CIED) recipients represents the most important expression of this technological revolution in the clinical electrophysiology field [1,2,3,4,5]. Nowadays, RM for CIED follow-up has been shown to be as effective and safe as in-person device follow-up and is recommended by current guidelines to reduce in-office visits and health-related costs [6]. 

Alongside RM, European and American guidelines also endorse yearly in-person visits and/or in-office evaluation for CIED recipients whenever clinically relevant RM alerts are recorded [6]. Beyond CIED recipients, clinical electrophysiology patients without CIEDs cannot be followed by RM, and usually need standard in-person visits. Coronavirus disease 2019 (COVID-19) restrictions, with the imposition of social distancing, have fueled the adoption of telemedicine as an alternative way to connect patients with their health care providers in an efficient and safe way. As a result, the use of virtual medicine has been empowered by a World Health Organization (WHO) declaration on 11 March 2020, to respond to the spread of the pandemic, leading to a novel reorganization of the health care system. Thus, during the COVID-19 pandemic, the RM coverage steeply increased and confirmed its role as a valuable tool in providing high-quality assistance to CIED recipients [7,8]. Simovic et al. [9] have recently reported the results of a European survey assessing the impact of the COVID-19 pandemic on RM of CIEDs. The survey suggested that the COVID-19 pandemic led to a significant increase in RM coverage among CIED recipients, with 65% of participants starting a new RM connection during the pandemic [9]. Similarly, a survey promoted by the Italian Association of Arrhythmology and Cardiac Pacing (AIAC) showed a progressive increase in the use of RM during the 2021 to 2017, followed by an exponential increase from 2017 to 2020, thus including the pandemic period [10]. Of note, 39% of participant centers described an increase > 30% in the number of CIED recipients followed by RM during COVID-19 outbreak [10]. Telemedicine use has been implemented in various clinical areas, including cardiology, especially with virtual visits (VVs). VVs are an important part of telecardiology services; and in recent years, in multiple clinical areas, VVs have been introduced as a new modality of delivering health care services in an efficient and cost-effective way [11]. More importantly, once it was established that COVID-19 was a systemic disease leading to a specific cardiovascular involvement and to a certain amount of cardiovascular side effects related to COVID-19 therapy [12,13,14], the use of VVs enabled the follow-up of COVID-19 patients after discharge, in addition to use among electrophysiology patients care facing a massive negative impact during the pandemic [8]. Multiple studies have addressed and validated the use of remote monitoring (RM) in CIED patients [15] but only small-scale studies have been reported in literature about patient satisfaction with the use of VVs, as well as the clinical impact on clinical electrophysiology patient management [16,17,18]. Evidence proving the feasibility, effectiveness and patient acceptance of the virtual modality of care as compared to the in-person modality of care in the clinical electrophysiology field would be pivotal in the widespread adoption of virtual visits. Therefore, the aims of our study were to evaluate the efficacy and feasibility of the virtual modality of care in the management of cardiac electrophysiology patients and patient satisfaction with VVs.

## 2. Materials and Methods

We performed a two-center prospective study including consecutive electrophysiology patients with and without CIED who were scheduled to undergo an in-office evaluation from 1 March 2021 to 31 August 2021 at the Department of Cardiovascular and Respiratory Diseases, Umberto I Hospital, Sapienza University of Rome and at the Cardiology Unit of Luigi Sacco University Hospital, Milan, Italy cardiac arrhythmia service clinics. In our experience, a VV was proposed to all CIED patients as an alternative to an already scheduled follow-up visit or in patients who presented RM cardiac arrhythmias and/or heart failure (HF) alerts. Moreover, a VV was also proposed to patients without CIED but referred to our center for overt or suspected arrhythmic problems, such as atrial fibrillation (AF), paroxysmal supraventricular arrhythmias, ventricular arrhythmias (VAs), and palpitations associated with other symptoms (pre-syncope, syncope or dizziness). The modality of the visit (VV or in-person visit) was chosen based on patient preference. VVs were remotely conducted using videoconferencing tools (smartphone, personal computer, and tablet) and using secured and locally approved institutional regional platforms.

The study population was divided into two groups: (1) patients who accepted the virtual modality of assistance; (2) patients who were unable to receive VVs due to technical constraints and received in-office visits. Technical constraints and issues were defined as: (1) absence or unreliability of internet connection; (2) unavailability of videoconferencing tools such as smartphone, personal computer, and tablet; (3) absence of technical skills to connect to the videoconferencing platform and/or unavailability of caregiver help to connect and complete VVs successfully. All patients who refused the VV modality due to their own preference and not for technical constraints were excluded from the present study to avoid a response bias.

The health care providers involved in the visits were the cardiology technician and the cardiologist.

As initials step, the technician checked visit indications, CIED reports and alerts of patients scheduled to receive an in-office evaluation during the study period. The cardiologist checked visit indications, reports and alerts collected by the technician; called the patient asking about the availability of videoconferencing tools, technical skills and/or caregivers able to help to connect and complete VVs successfully. For patients able to receive a VV, the cardiologist dialed by phone, proposing a VV; and if the patient accepted the virtual modality of care, specific informed consent was acquired. Then, the cardiologist prescribed and booked the visit using the regional VV platform, and sent the patient the appointment confirmation, using an institutional e-mail address and eventually prescribed and booked further exams, such as blood tests, electrocardiograms (ECG), and transthoracic echocardiograms (TTE), asking the patient to send the reports to the institutional e-mail address the day before the scheduled visit. Moreover, the cardiologist asked the patient to send all prior clinical records/documents (prior ECG, TTE, cardiac magnetic resonance, Holter monitoring, etc.), the day before the VV.

At the time of the VV, the health care provider connected to the regional platform using the link on the prescription shared only with the patient. During the VV, the cardiologist asked for any HF-related or arrhythmia-related signs and/or symptoms, vital parameters, and further clinical information; at the end of the VV, the cardiologist asked the patient to evaluate his/her experience through a questionnaire consisting of 18 questions and sent the reports of the visit to the patient using institutional e-mail address. Specifically, the following data were collected during VVs and in-office visits: blood pressure (BP), heart rate (HR), oxygen saturation (SO), body weight, medications, clinical events and symptoms. Moreover, visit duration was noted, although some technical data were recorded only for the virtual modality, such as the setup connection duration, the overall VV duration and the connection quality. Connection quality was rated as poor if frequent audio and/or video interruption made the VV impossible, and good if only short interruptions of audio and/or video signals occurred, without hampering the communication among patients and the cardiologist. The connection quality was rated excellent if no interruptions of audio/video signals occurred during the VV.

At the end of the visit, both VVs and in-office visits, patients were asked to complete the Short-Form Patient Satisfaction Questionnaire (PSQ-18) to evaluate patient satisfaction [19]. Patients of the in-person group received and completed the questionnaire in the office, while patients in the VV group received the questionnaire after the VV and were asked to send the questionnaire by e-mail to the cardiologist the same day of the visit. The PSQ-18 is derived from the 80-item Patient Satisfaction Questionnaire (PSQ), and examines global satisfaction with medical care including 7 specific aspects/subscales: general satisfaction (items 3 and 17); technical quality (items 2, 4, 6, and 14); interpersonal manner (items 10 and 11); communication (items 1 and 13); financial aspects (items 5 and 7); time spent with doctor (items 12 and 15); accessibility and convenience (items 8, 9, 16, and 18). Some PSQ-18 items are worded so that agreement reflects satisfaction with medical care, whereas other items are worded so that agreement reflects dissatisfaction with medical care. All items were scored so that high scores reflected satisfaction with medical care. After the item scoring, all items within the same subscale were averaged together to create 7 subscale scores. The outcome of interest evaluated in the present study was the feasibility and effectiveness of VVs, as compared to in-person visits, which was measured as: (1) improvement in symptoms after the index visit, (2) disappearance of remote monitoring alerts at follow-up, (3) necessity of urgent hospitalization and (4) patient satisfaction measured by the PSQ-18.

### Statistical Analysis

Continuous data were expressed as the mean and standard deviation. Categorical variables were expressed as numbers and percentages. Between-group comparisons were performed using Student’s *t*-test for continuous variables and the Chi Square test for categorical variables. Statistical analysis was performed using SPSS statistical software (release 26.0; SPSS Inc., Chicago, IL, USA). A *p* value ≤ 0.05 was considered statistically significant.

## 3. Results

### 3.1. Demographic Characteristics

Among 6701 patients followed in the enrolling centers, 74.8% (5011 patients) were CIED recipients and 1456 (29.6%) patients were followed by RM at the time of study inception. During the study period, 492 visits were performed, divided as 162 VVs and 330 in-office visits. Among 330 patients who have undergone an in-office visit, 185 were not able to perform VVs due to technical issues or lack of caregiver help; the last 145 patients declined the virtual modality of assistance and were excluded from the current study. Hence, the final study population included 162 (46.7%) patients in the VV group and 185 (53.3%) in the in-office visit group.

The mean age of the study population was 70.2 ± 13.5 years and 209 (60.2%) were males. No differences were found in demographic baseline characteristics among patients who received VVs versus in-office visits (Table 1). Of note, 61 out 162 patients of the VV group (37.6%) had their first-ever clinical electrophysiology visit as a VV. Among patients who received VVs, 67 (41.4%) were not CIED recipients, 52 (32.1%) had an implantable loop recorder (ILR), 26 (16%) had an implantable cardioverter defibrillator (ICD) or cardiac resynchronization therapy (CRT) and 17 patients (10.5%) had a pacemaker (PM). In 40.9% of cases (142 patients), the follow-up visit was related to an arrhythmic alert detected by the RM, whereas clinical evaluation related to arrhythmia-related symptoms was performed in 76 patients (21.9%). In 55 cases (15.8%), the prescription of novel oral anticoagulants (NOACs) was the reason for follow-up, while routine CIED follow-up was performed in 28 patients (8.1%). In both groups, AF detection represented more than a half of total arrhythmic alerts, while bradyarrhythmic and ventricular events were recorded in 14 and 29 patients, respectively. In 10 patients, the visit was related to a fluid monitoring alert indicating fluid overload and potentially incipient heart failure (Table 1).

### 3.2. Visit Data

The following vital parameters were investigated during any visit: weight, blood pressure, heart rate, and oxygen saturation (Table 2). In particular, data about BP, HR and oxygen saturation were obtained in 93%, 95% and 100% of patients during in-person visits, and in 91%, 90%, 93% of VV patients, respectively. Cardiologists investigated patient symptoms during evaluation. In the VV group, 67.3% of patients were asymptomatic, 28.4% complained of dyspnea or palpitations and only 4.3% presented with fatigue/pre-syncope. No difference in symptom presentation was found among the VV and in-office visit groups (Table 2).

### 3.3. Technical Aspects of Virtual Visits

Of the patients included in the VV group, 85 (52.5%) used a smartphone for the VV, 51 (31.5%) used a personal computer, and 26 (16%) used a tablet. The mean setup duration was 5.93 ± 4.31 min, while the mean VV duration was 21.9 ± 5.56 min and the mean total connection duration including setup and the VV was 27.8 ± 7.8 min. One visit was not conducted due to poor connection quality. No difference was found in the mean visit duration among the two groups (Table 2). Connection quality was rated as poor in only 1 case (0.6%), while it was rated as good in 48.1% of cases and as excellent in 51.2% of VVs. Patients were helped by a caregiver in 92 cases (56.8%), and the caregiver was the patient’s relative in 91.3% (*n* = 84) of cases. In 38.9% of cases (63 patients), the videoconferencing tool belonged to the patient’s relative.

### 3.4. The PSQ-18 Results and Patient Preference

**Figure 1:**
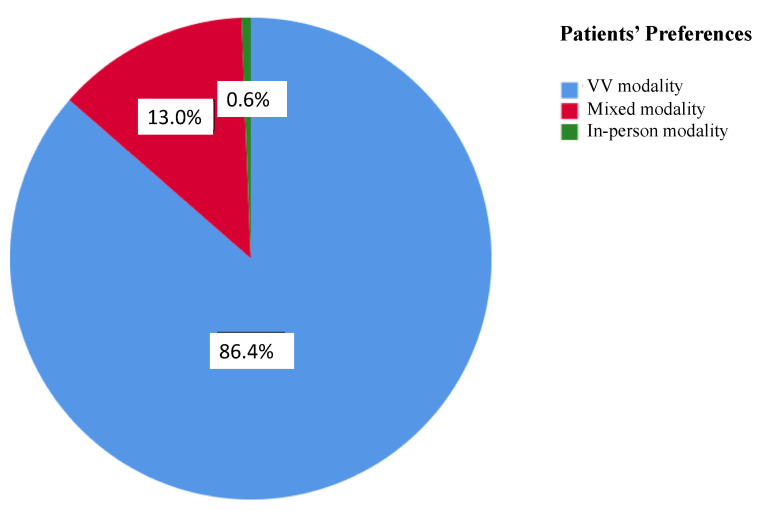
Patient preferences for each visit modality after the COVID-19 pandemic is over. Responses are expressed with different colors and percentages.

**Figure 2:**
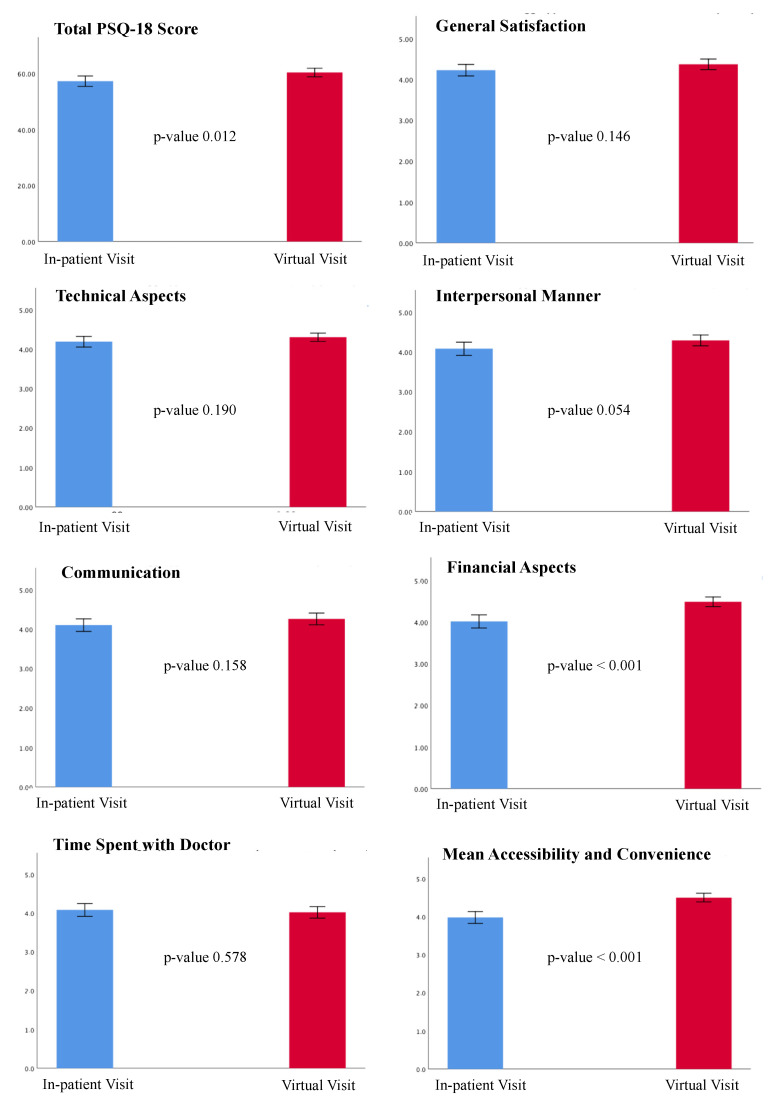
Results from the PSQ-18. The total PSQ-18 score and the scores for the seven subscales are shown. Results are expressed as the means and standard deviations. Blue color represents the in-patient group; red color represents the virtual visit group. The *p*-value for each comparison is shown. A *p*-value < 0.05 was considered statistically significant. PSQ-18: Patient Satisfaction Questionnaire-18.

The overall PSQ-18 scores and subscales are listed in Table 3. Of note, 90.1% patients (146) of the VV group and all but 5 patients (97.3%, 180) of the in-person visit group completed the survey. A statistically significant difference was found in the total PSQ-18 scores between the 2 groups (*p*-value 0.012), with higher satisfaction associated with VVs (Figure 1). VVs were related to higher ratings in the subscales of financial aspects (*p*-value < 0.001) and accessibility and convenience (*p*-value < 0.001), whereas there was a trend favoring VVs in the subscale of interpersonal manner, although this difference did not reach statistical significance (*p*-value 0.054). Further, 140 out 162 patients (86.4%) indicated that they would prefer a virtual visit over an in-person visit even after the COVID-19 pandemic, 21 patients (13%) would prefer a mix of in-person visits and VVs, and only 1 patient (0.6%) would prefer an in-person visit (Figure 2). However, this patient did not undergo a VV due to a technical issue (poor connection quality) and was scheduled to receive an in-person evaluation.

### 3.5. Feasibility and Safety of Virtual Visits

Following the clinical evaluation, no changes in medical therapy were made in 143 (41%) patients, while medical therapy was changed by the cardiologist in 21% of cases according to patient status. Of note, no differences were found in the management of patients among the study groups, with 19.8% of patients undergoing drug therapy adjustments in the VV group and 22.2% in the in-office group (*p*-value 0.582). In particular, among 32 patients in the VV group, the diuretic dose was adjusted for 7 patients, the beta-blocker dose for AF rate control and/or for NSVT was adjusted for 15 patients, 8 patients were started on bisoprolol, 5 patients were started on amiodarone for NSVT or paroxysmal AF, and anticoagulant therapy was prescribed for 3 patients. Among 41 patients in the in-person group, the loop diuretic dose was up-titrated for 5 patients, spironolactone was added for 2 patients, the beta-blocker dose was increased for 25 patients or they were started on a beta-blocker, 7 patients were started on amiodarone and 4 patients were started on anticoagulant therapy for the onset of AF. In 30% of patients, further examinations and analysis were prescribed (echocardiogram in 55% of cases, chest X-ray in 20% of cases, Holter ECG in 20% of cases, and event recorder in 5% of cases); and in 8% of cases, hospitalization (either urgent or programmed) was recommended (Table 2). In this latter group, eight VV patients were scheduled for programmed hospitalization: two patients for incipient HF with symptoms in spite of un-titration of diuretic drug dose, one patient for ICD generator replacement, two patients for ILR implantation (one patient had syncope and one patient pre-syncope and dizziness), three patients for PM implantation due to the evidence of II degree II type sino-atrial block (in two patients, recorded by Holter-ECG) and transient II degree type I atrio-ventricular block (AVB) recorded at ILR and associated with dizziness in one patient. Similarly, in the in-person group, programmed hospitalization was scheduled for 11 patients: 3 patients with worsening HF, 2 PM generator replacements, 1 PM implant revision with atrial lead repositioning after phrenic nerve stimulation evidence, 2 PM implantation for sinus pauses at ILR recordings and 3 ILR implantation for syncope of unknown cause and normal basal EKG. Further, urgent hospitalization was required for eight patients: four patients in the VV group (two patients with appropriate ICD shocks and two patients with III-degree AVB associated with syncope) and four patients in the in-person group (one patient with ICD lead fracture associated with inappropriate electric storm and three patients with III degree AVB associated with symptoms).

After a mean follow-up of 6 ± 3.8 months, there was a 47.2% reduction in RM alerts as compared to baseline RM alerts. In particular, 75 alerts were recorded at follow-up, without any difference between the in-person visit modality and the VV modality (42 versus 33, *p*-value 0.598). Of note, relative reductions in RM alerts at follow-up among the in-person visit group and the VV group were 43.2% and 51.5%, without any statistically significant difference (*p*-value 0.527). At follow-up, 36 (10.4%) patients still complained of arrhythmia or HF-related symptoms—14 from the VV group and 22 from the in-person visit group (*p*-value 0.321). No difference was found in the relative reduction in symptomatic patient rates among the two groups (73.6% in the VV group and 56.9% in the in-office group, *p*-value 0.073).

Lastly, eight patients (2.3%) required urgent hospitalization, two patients for appropriate ICD shocks due to sustained VAs and six patients for the presence at ILR recording of advanced AVB associated with pre-syncope. No differences were found in urgent hospitalization rates among the study groups (*p*-value 0.849). No deaths were observed in both groups (Table 2).

## 4. Discussion

This study describes our experience with the virtual modality of assistance for cardiac electrophysiology patients, with and without CIED. Our research assessed the clinical impact of VVs on a specific population of clinical electrophysiology patients and evaluated patient satisfaction with the virtual modality of care.

The major findings of our study are:

- VVs are feasible in managing patients with arrhythmic disorders, either as a first visit or as a follow-up visit; the mean VV setup duration was 5.93 ± 4.31 min and we did not find significant differences in the mean duration between VVs and or in-person visits.
- Adjustment in medical therapy was feasible in both study groups, without any need for switching the visit modality from a virtual to an in-person setting to obtain a clinical benefit.
- VVs seem as effective as in-person visits, resulting in a similar reduction in RM alerts (51.5% vs. 43.2%, *p*-value 0.527) and in symptomatic patient rates (73.6% vs. 56.9%, *p*-value 0.073) at follow-up, without differences in urgent hospitalization rates.
- Patient satisfaction with the virtual modality of assistance was high, as VVs outperformed the in-person modality in the subscales of financial aspects (*p*-value < 0.001) and accessibility and convenience (*p*-value < 0.001).

### 4.1. Feasibility of Virtual Visits: Are We Ready for Remote Health Care Delivery?

The COVID-19 pandemic has been a great challenge for the health care system all over the world and has fueled a reorganization of outpatient care, accelerating the transition from routine clinic follow-up to remote health delivery, to reduce the contagion risk for patients and health care providers, though maintaining a high standard of care. Indeed, the adoption of telemedicine, defined as the use of medical information exchanged from one site to another through remote communication to improve patient health, has risen steeply from 11% in 2019 to 46% currently in the USA [20]. Meanwhile, guidance statements in the electrophysiology and HF fields recommended a change to telehealth, leveraging data collected via RM from CIED to remotely manage patients [7,8]. In a retrospective cohort study comparing outpatient cardiovascular visit volumes pre-COVID-19 versus in the COVID-19 period, Kalwani et al. [21] reported an increase in telecardiology use from 3.5% to 63%. Of note, telemedicine use peaked above 75% in all cardiovascular subspecialties during the COVID-19 outbreak, but only clinical electrophysiology maintained a high rate of the virtual modality of care (over 95%) [21]. In our experience, the COVID-19 outbreak prompted the adoption of RM programs in all patients with CIED who were eligible for a RM strategy, with either at-home delivery or in-office modem pick-up, to provide easier access to valuable information, such as arrhythmic events, acute HF manifestations and device-related issues, without the need for in-person CIED interrogation [22,23,24,25,26].

In this analysis, we showed that the virtual modality of health care delivery is feasible among clinical electrophysiology patients, and only one VV was not conducted due to poor connection quality; no difference in total visit duration among the VV group and the in-person group was found. The lack of relevant differences in the mean visit duration may be related to the organization underlying VVs; indeed, the health care providers involved in VVs systematically received all the medical reports related to followed-up patients the day before the VV, thus orienting VV purposes, preparing NOACs or further exams prescription in advance, and thereby sparing time during the videoconference.

Moreover, we reported a relatively short mean setup time of 5.93 ± 4.31 min, with more than a half of patients using a smartphone as the chosen videoconferencing tool. Of note, 56.8% of VVs were assisted by a caregiver; and in more than 1/3 of cases, the videoconferencing tool belonged to the caregiver. Hence, a virtual modality of assistance is feasible, but it significantly relies on caregivers’ technical assistance; and in 38.9% of cases, on caregivers’ tools. This evidence may be explained by the relatively old age of our clinical electrophysiology population. For instance, the prevalence of AF and conduction system diseases requiring CIED both increase with population aging, while technological skills, internet coverage and tolls as smartphones, tablets or personal computers are generally less available in the elderly. Thus, the presence of caregivers becomes pivotal to allow the adoption of telemedicine as a standard-of-care system. Indeed, nowadays, technological limitations represent the major drawback of the virtual modality of assistance, as underscored by the large number of patients (*n* = 185, 53.3% of the study population) that could not undergo VVs due to technical constraints and/or absence of technical assistance. In this regard, a recent Canadian study reported the results of three surveys, conducted after VVs, exploring the perspectives of patients, caregivers and health care providers, addressing different fields such as the use of technology, experience with VVs and preferences for future visits [17]. Of note, 77% of visits were conducted by telephone and only 23% by videoconference. Although 68% of patients and caregivers had previously participated in at least 1 video call for any purpose, 28% of respondents who had a telephone visit indicated that technology would have been a concern or a significant challenge if they had had a visit by a videoconference system. Hence, also in a population experienced with videoconferencing tools, technological aspects still represent a significant barrier. In this view, our study sheds some light on the need to invest economical and social resources for the improvement of technical support, as well as widespread availability of high-speed internet connection, to provide basic requirements for digital distribution of medicine.

### 4.2. Clinical Outcomes Associated with the Virtual Modality of Care

Feasibility of VVs was not only evaluated in technological terms but also in clinical terms as changes in the patient diagnostic and therapeutic pathway after the visit. Indeed, the feasibility of VVs strictly depends on the possibility of health care providers to manage patients as they would manage them in the in-person setting, thus changing medical therapy, scheduling further exams, intervention or hospitalizations [27]. Santini et al. [28] published a prospective multicenter registry evaluating a protocol for RM of HF patients using the HeartLogic algorithm. They showed that HF patients management via RM was feasible, with 90% of HeartLogic alerts triggering clinical actions, mainly diuretic dosage increase, other drug adjustment or HF hospitalization [28]. A recent study conducted on 43 patients with HF and CRT undergoing VVs showed that decision making after a VV was feasible, with findings on virtual examination leading to further exams, medical changes and interventions such as up-titration of drugs or lead extraction [16]. However, no comparison in terms of decision making between the VV group and the in-person group was reported. Conversely, we did not find any differences in the clinical management of patients among the two study groups, with similar percentages of patients undergoing changes in medical therapy (*p*-value 0.582), further exams prescription (*p*-value 0.715) and programmed hospitalizations (*p*-value 0.680). Our results are in line with those recently published by Calò et al. [29] on HF patients remotely managed using the HeartLogic algorithm. Calò et al. showed that 75% of incipient HF decompensation RM alerts were remotely managed and did not require an in-person visit [29]. Moreover, the study did not show differences in HF event probability reduction among patients remotely managed and patients followed by in-office visits (HR 0.99, 95% CI 0.37–2.68, *p*-value 0.993), underscoring the possibility of managing HF patients without increasing the clinic workload in terms of in-person visits.

In our study, delivery of care with a virtual modality has been proven to be as effective as an in-person modality of assistance, resulting in a similar reduction in the number of RM alerts (51.5% vs. 43.2%, *p*-value 0.527) and in the rate of symptomatic patients (73.6% vs. 56.9%, *p*-value 0.073) during follow-up. Moreover, similar urgent hospitalization rates were observed (2.5% in the VV group vs. 2.2% in the in-person group), without any statistically significant difference (*p*-value 0.849). No deaths were observed in both groups during the study period. Our study confirmed and expanded the results of a previous small study [16] on HF patients with CRT, showing no differences in a combined outcome of all-cause mortality and HF or device-related hospitalizations between the in-person visit group (*n* = 39) and the VV group (*n* = 43), during a short follow-up period of 82 [61–96] days. More recently, Alhejily reported a prospective cohort study of a telecardiology clinic using video-assisted chatting as a means of communication, evaluating hospital admissions and emergency visit rates [30]. Over a 10 month period, 277 patients underwent a video-assisted visit, mainly complaining of chest pain, palpitations and uncontrolled systemic arterial hypertension. Drug and new investigation prescriptions were performed in 49% and 58% of cases, respectively. Compared to an in-office visit, video-assisted chatting was associated with significantly higher rates of hospitalizations and emergency visits, mainly driven by ischemic heart disease needing revascularization and decompensated HF, whereas no difference in terms of death rate was found among the two groups of patients [30]. As previously reported, we did not find any difference in hospitalization rates and mortality rates among the two groups of clinical electrophysiology patients, and we showed that the adoption of a VV modality of care is associated with similar benefits in terms of symptom relief and RM alert reductions as compared to the in-person group.

### 4.3. Remote Delivery of Care and Patient Satisfaction

Telemedicine aims to improve the patient’s experience of care, empowering patients to participate in disease management in shared decision making with health care providers [31,32]. In this setting, patient preferences and satisfaction with VVs are crucial to evaluate the acceptance and success of a telehealth system. In this study, we reported patient satisfaction using the PSQ-18, and, for the first time ever, we compared satisfaction associated with VVs versus in-person visits. We found higher satisfaction associated with VVs, with a statistically significant difference in the total PSQ-18 scores between the two groups (*p*-value 0.012). Indeed, the health care virtual modality was related to higher ratings in the subscales of financial aspects (*p*-value < 0.001) and accessibility and convenience (*p*-value < 0.001), as compared to the in-person modality. Moreover, we reported patient preferences for the next visits: 140 out 162 patients (86.4%) indicated that they would prefer a VV over an in-hospital visit as the follow-up modality after the COVID-19 pandemic, while 21 patients (13%) would prefer a mix of in-person and VV follow-up. These results are in line with recently reported studies using surveys to evaluate patient satisfaction with VV. Zhao et al. [16] recently reported patient satisfaction with a virtual multidisciplinary care model for HF patients with CRT during the COVID-19 pandemic. Among 43 patients in the VV group, 21 answered survey questions: >90% reported either very satisfied or satisfied with telemedicine use and 100% of patients would like to use VVs again. Hu et al. [18] performed a prospective survey of cardiac electrophysiology patients undergoing VVs, reporting patient overall level of satisfaction as excellent or very good in 98.4% of cases. Sanderson et al. [17], despite limited available data (45 patients, 2 caregivers and 26 health care providers completed the surveys), showed that 88% of patients and caregivers were satisfied with the virtual format of the visit, and only 9% were dissatisfied. Moreover, 72% of participants would prefer primarily VVs or a mix of in-person visits and VVs after the COVID-19 pandemic is over [17]. Interestingly, patients and caregivers were asked to pinpoint factors considered concerns or challenges when attending in-person visits: 36% of respondents included availability of transportation, 34% cost of transportation, 40% time for in-person visits and 40% infection control. These answers pointed out that, from a patient and caregiver points of view, the reduction in contagion risk due to a potential exposure to COVID-19 is as important as the reduction in economic and social burdens related to the modality of health care delivery. The PSQ-18 results in our study are in line with this concept: the virtual modality of care outperformed in-person visits in the subscales of financial aspects and accessibility/convenience, clearly highlighting the strengths of VVs. Our results confirm the economic benefits related to RM for the follow-up of CIED recipients. In particular, the Health Economics Evaluation registry for Remote Follow-up (TARIFF study) was designed to assess the costs and the benefits of RM as compared to the standard of care (SC) from the perspectives of the health care system, patients and caregivers [33]. The overall mean annual cost per patient in the SC was significantly higher than in the RM group (*p*-value < 0.001), with a reduction in costs of 53.8% in the RM group mainly driven by the reduction in the costs related to cardiovascular (CV) hospitalizations (*p*-value 0.003). Similarly, the MORE-CARE randomized controlled trial [34] showed a significant 38% reduction in the composite endpoint of health care resource utilization (including CV hospitalizations, CV emergency department admissions, and CV in-office scheduled or unscheduled follow-ups) for 437 CRT-D patients followed by remote checks alternated to in-office follow-ups as compared to 428 CRT-D patients followed by in-person visits alone (incidence rate ratio 0.62, 95% CI 0.58–0.66, *p*-value < 0.001).

Our study confirms that the virtual modality of care may represent a feasible and effective approach in clinical electrophysiology patient management, with favorable overall patient satisfaction, and supports the post-pandemic sustainability of a virtual care model for these patients. Although new evidence should be awaited, the encouraging results of the virtual modality of health care delivery, alongside with the growing investment of economical, technical and social resources in this field, will probably result in the persistence of VVs beyond the COVID-19 pandemic, in a new mixed model of care, in which a VV guarantees a regular and strict follow-up and helps the cardiologist to decide the correct timing of in-person visits.

### 4.4. Limitations

Our study presents several limitations. First, this is a two-center, observational study, with a relatively small sample size. Hence, our results need to be confirmed in larger, randomized studies. Second, the presence of a response bias affecting the PSQ-18 results cannot be excluded. However, we proposed VVs to all patients and those who refused the VV modality due to preference and not for technical constraints were excluded from the present study to avoid this response bias. On the other side, it should be noted that this may have led to a selection bias, especially regarding patient satisfaction assessment. Third, our study had a relatively short follow-up time. Further, we did not report health care providers’ preferences that could have added some suggestions on how to improve VV service.

## 5. Conclusions

Our study suggests that the virtual modality of health care delivery is feasible and effective for cardiac electrophysiology patient follow-up. No significant differences were found in clinical outcomes when comparing this remote management strategy to a traditional in-person visit modality. Patients overall preferred VVs over in-person visits mainly for financial aspects, accessibility, and convenience of this strategy. In the future, a hybrid health care system including virtual and in-person modalities will probably represent the new standard of care, allowing improvements in cardiac electrophysiology patient management and follow-up.

## Figures and Tables

**Table 1:** Baseline population characteristics.

Demographics	Total Population (*n* = 347)	Group Virtual Visit (*n* = 162)	Group In-Person Visit (*n* = 185)	*p*-Value
**Age, year**	70.2 ± 13.5	69 ± 12.7	71 ± 14.1	0.648
**Male, *n* (%)**	209 (60.2%)	99 (61.1%)	110 (55.8%)	0.09
**CIED**	209 (60%)	95 (58.6%)	114 (61.1%)	0.571
ICD, *n* (%)	52 (15%)	20 (12.3%)	32 (17.3%)	
CRT, *n* (%)	14 (4%)	6 (3.7%)	8 (4.3%)	
PMK, *n* (%)	39 (11.2%)	17 (10.5%)	22 (12%)	
ILR, *n* (%)	104 (30%)	52 (32.1%)	52 (28%)	
No CIED, *n* (%)	138 (40%)	67 (41.4%)	71 (62.3%)	0.571
**Heart Disease**				
IHD, *n* (%)	111 (32%)	45 (27.8%)	66 (35.6%)	0.115
VHD, *n* (%)	45 (13%)	21 (13%)	24 (13%)	0.997
Channelopaties, *n* (%)	6 (1.7%)	2 (1.2%)	4 (2.2%)	0.335
Others, *n* (%)	17 (5%)	7 (4.3%)	10 (18.5%)	0.640
**LVEF, %**	46 ± 12	44 ± 11	45 ± 12	0.810
**Comorbidities**				
Diabetes mellitus, *n* (%)	118 (34%)	57 (35.2%)	61 (32.9%)	0.664
COPD, *n* (%)	59 (17%)	26 (16%)	33 (17.8%)	0.658
Hypertension, *n* (%)	198 (57%)	92 (56.8%)	106 (57.3%)	0.924
Chronic kidney disease, *n* (%)	76 (22%)	35 (21.6%)	41 (22.2%)	0.900
**Visit setting**				
RM Alert *n* (%)	142 (40.9%)	68 (42%)	74 (40%)	0.708
Arrhythmologic visit *n* (%)	76 (21.9%)	35 (21.6%)	41 (22.2%)	0.900
NOAC prescription *n* (%)	55 (15.8%)	28 (17.3%)	27 (14.6%)	0.288
CIED follow-up *n* (%)	28 (8.1%)	12 (7.4%)	16 (8.6%)	0.671
**Type of RM alert**				
Atrial fibrillation *n* (%)	82 (57.7%)	37 (54.4%)	43 (58.1%)	0.929
ICD therapy *n* (%)	3 (2.1%)	2 (2.9%)	1 (1.3%)	0.485
Ventricular arrhythmias *n* (%)	29 (20.4%)	18 (26.5%)	11 (14.9%)	0.082
Bradyarrhythmias *n* (%)	14 (9.9%)	5 (7.4%)	9 (12.2%)	0.336
Fluid monitoring alert	10 (7%)	5 (7.4%)	5 (6.8%)	0.889
Device-related malfunction *n* (%)	4 (2.8%)	1 (1.5%)	3 (4%)	0.381

CIED: cardiac implantable electronic device; CRT: cardiac resynchronization therapy; COPD: chronic obstructive pulmonary disease; ICD: implantable cardioverter defibrillator; IHD: ischemic heart disease; ILR: implantable loop recorder; LVEF: left-ventricle ejection fraction; NOAC: novel oral anticoagulants; PM: pacemaker; RM: remote monitoring; VHD: valvular heart disease.

**Table 2:** Vital parameters, visit duration, presenting symptoms, RM alerts and decision after visit and RM and symptoms at follow-up. RM: remote monitoring.

	Total Population (*n* = 347)	Group Virtual Visit (*n* = 162)	Group In-Person Visit (*n* = 185)	*p*-Value
**Variable Investigated**				
Body weight (kg)	83.5 ± 18.95	82.5 ± 18.9	84.6 ± 19.8	0.146
Systolic blood pressure (mmHg)	133.8 ± 23.6	136.4 ± 23.5	131.9 ± 22.7	0.547
Diastolic blood pressure (mmHg)	78.6 ± 13.5	77 ± 13.2	79.6 ± 13.6	0.248
Heart rate (bpm)	73.3 ± 9.43	73.7 ± 9.74	72.6 ± 8.9	0.659
Oxygen Saturation (%)	98.8 ± 0.67	98.19 ± 0.84	99 ± 0.78	0.170
**Mean visit duration (min)**	22 ± 5.45	21.9 ± 5.56	22.2 ± 5.37	0.588
**Symptoms**				
Asymptomatic	243 (70%)	109 (67.3%)	134 (72.4%)	0.296
Dyspnea	38 (11%)	16 (9.8%)	22 (11.9%)	0.548
Palpitations	52 (15%)	30 (18.6%)	22 (11.8%)	0.084
Fatigue	3 (0.8%)	1 (0.62%)	2 (1.1%)	0.641
Pre-syncope	11 (3.2%)	6 (3.7%)	5 (2.7%)	0.595
**Decision after visit**				
No changes in medical therapy	143 (41%)	71 (43.8%)	72 (38.9%)	0.354
Changes in medical therapy	73 (21%)	32 (19.8%)	41 (22.2%)	0.582
Further examination prescribed	104 (30%)	47 (29%)	57 (30.8%)	0.715
Programmed hospitalization	19 (5.7%)	8 (4.9%)	11 (5.9%)	0.680
Urgent hospitalization	8 (2.3%)	4 (2.5%)	4 (2.2%)	0.849
**RM alert at follow-up**	75 (21.6%)	33 (20.4%)	42 (22.7%)	0.598
**Symptoms at follow-up**	36 (10.4%)	14 (9.9%)	22 (11.9%)	0.321

**Table 3:** Results from the PSQ-18.

PSQ-18 Scale	Group Virtual Visit (*n* = 162)	Group In-Person Visit (*n* = 185)	*p*-Value
Total Score	60 ± 9.9	57.4 ± 12.9	**0.012**
General Satisfaction	4.37 ± 0.84	4.22 ± 0.97	0.146
Technical Aspects	4.30 ± 0.68	4.19 ± 0.92	0.190
Interpersonal Manner	4.29 ± 0.88	4.08 ± 1.13	0.054
Communication	4.25 ± 0.96	4.10 ± 1.1	0.158
Financial Aspects	4.48 ± 0.75	4.01 ± 1.1	**<0.001**
Time Spent with Doctor	4.02 ± 0.96	4.1 ± 1.13	0.578
Accessibility and Convenience	4.5 ± 0.73	3.98 ± 1.07	**<0.001**

The total PSQ-18 score and the scores for the seven subscales are shown. Results are expressed as the means and standard deviations. The *p*-value for each comparison is shown. A *p*-value < 0.05 was considered statistically significant. PSQ-18: Patient Satisfaction Questionnaire-18.

## Data Availability

The data presented in this study are available on request from the corresponding author. The data are not publicly available due to privacy restrictions.
